# Segmental distribution and influencing factors of thoracolumbar osteoporotic vertebral compression fracture

**DOI:** 10.1186/s13018-026-06799-z

**Published:** 2026-03-16

**Authors:** Xinyue Zheng, Bingyan Wang, Hehu Tang, Jiaxin Fu, Jiesheng Liu, Shizheng Chen, Shujia Liu, Fangyong Wang, Junwei Zhang, Zhen Lyu

**Affiliations:** 1 School of Rehabilitation, Capital Medical University, Beijing 100068, China; 2 Department of Spine and Spinal Cord Surgery, Beijing Bo’ai Hospital, China Rehabilitation Research Center, Beijing 100068, China

**Keywords:** Osteoporosis, Osteoporotic vertebral compression fracture, Percutaneous vertebroplasty, Recurrent fracture, Spine alignment, CT value, Bone cement distribution

## Abstract

**Background:**

Percutaneous vertebroplasty (PVP) is highly effective treatment for osteoporotic vertebral compression fractures (OVCF). There is no clear conclusion regarding the common locations of fractures, and whether recurrent fractures are related to surgery, or changes in local spinal alignment.

**Methods:**

A total of 164 patients with thoracolumbar OVCF from June 2020 to June 2024 were enrolled. The segmental distribution with initial and recurrent fractures were collected. The impact of surgery on recurrent fractures was analyzed by comparing the segmental distribution of recurrent fractures between groups. 84 patients with T11-L2 OVCF were divided into two groups based on recurrent fractures or not. The gender, age, trauma of patients were measured, imaging indicators including bone cement distribution type, T11-L2 Cobb angle, Cobb angle and height recovery rate of fractured vertebra, and vertebral CT value were measured at the time of initial fracture, immediately after surgery, and recurrent fracture. The influencing factors of recurrent OVCF were explored through inter-group comparative analysis.

**Results:**

Both initial and recurrent fractures mostly occurred in the T11-L3, accounting for 80.4% and 63.1%, respectively. In the non-surgical group, the recurrent fractures were found mostly at L1 and L2 levels, accounting for 21.6%; while in the surgical group, the fractures occurred mostly at L1 level, accounting for 14.0%. In 84 patients with T11-L2 OVCF, among surgical patients, average age in the recurrent fracture group was 8.4 years older(*P*<0.001), and the average CT value was 22.9 HU lower (*P* =0.012) than that in the non-recurrent group. For each 1-year increase in age, the risk of recurrent fractures increased by 7.1%; for each 1 HU decrease in CT value, the risk of recurrent fractures increased by 2.1%. In addition, 78.6% of patients with initial fractures had a history of trauma, and 64.7% of patients with recurrent fractures had a history of trauma.

**Conclusions:**

Initial and recurrent OVCF of the thoracolumbar spine commonly occurred in T11 to L3 segments. The segment of initial fracture and surgical treatment were not associated with recurrent fracture location or risk. Advanced age and decreased CT value are risk factors for recurrent fractures. The occurrence of the fracture, mostly depends on the presence of trauma.

**Registry:**

www.chictr.org.cn, TRN: ChiCTR2500105987, Registration date: 27 June 2025.

## Background

Osteoporotic vertebral compression fracture (OVCF) is one of the most common complications of osteoporosis, which is characterized by severe low back pain affecting daily activities and reducing the quality of life. A multinational epidemiologic study found that the prevalence of vertebral fracture in China was 14.3% in men aged 60–69 years, and 15.5–22.6% in women, and the incidence rate of spinal fracture in urban areas increased rapidly [1]. This type of fracture is characterized by high morbidity, underdiagnosis and high incidence of recurrence [2–6]. Percutaneous vertebroplasty (PVP) and percutaneous balloon-expandable kyphoplasty (PKP), as the most commonly used treatments, can provide rapid and effective pain relief and improve quality of life [7–10]. However, there is currently no clear conclusion on whether recurrent fractures are related to the treatment method of the initial fracture, or to changes in the local spinal alignment, etc. [6, 11–13]. The purpose of this study is to investigate the segmental distribution of thoracolumbar OVCFs, including initial fractures and recurrent fractures. Meanwhile, it aims to analyze the effects of factors such as trauma, age, CT value, and spinal alignment parameters on the occurrence of fractures.

## Methods

### General information

A total of 164 patients were selected from 611 patients with thoracolumbar osteoporotic vertebral compression fractures (OVCF) who visited our hospital from June 2020 to June 2024. Informed consent to participate was obtained from all of the participants. The inclusion criteria were as follows: (1) Aged 55–94 years old, diagnosed with osteoporotic vertebral compression fractures; (2) Fracture segments ranging from T1 to L5, with complete imaging data; (3) Newly occurred compressive fractures confirmed by CT and MRI. The exclusion criteria were: (1) Spinal tumors; (2) Lack of necessary information in medical records; (3) Pathological fractures caused by spinal infections.

### Evaluation criteria and methods

**Fig. 1:**
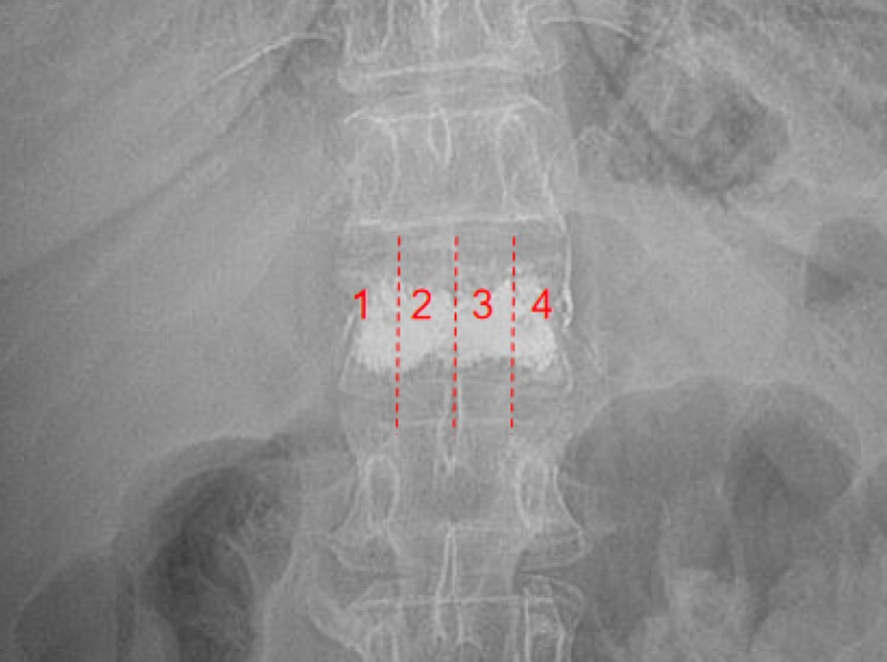
Zoning method for bone cement distribution on AP views

Information on whether patients had a history of trauma at the time of fracture (including carrying objects, bending over, sprains, falls, etc.) was collected through telephone follow-ups and medical record reviews. The segmental distribution of initial and recurrent fractures in 164 patients was investigated by studying imaging data. Then, the 164 cases were divided into three groups based on whether surgical treatment was received after the initial fracture and whether recurrent fractures occurred: the surgical-recurrent fracture group (78 cases), the non-surgical-recurrent fracture group (29 cases), and the surgical-non-recurrent fracture group (57 cases), so as to study the impact of surgery on recurrent fractures. Recurrent fractures confirmed by CT/MRI, occurring ≥ 1 month after the initial fracture of a non-identical vertebral body. Surgical patients underwent PVP or PKP treatment, while those receiving conservative treatment were instructed to rest in bed and wear a brace. All patients were guided in limb rehabilitation exercises and were prescribed regular anti-osteoporosis medications, including calcium supplements, vitamin D, bisphosphonates, and other relevant drugs. Next, focusing on 84 cases of T11-L2 OVCF among the above cases, a further analysis of the factors influencing fractures was conducted. The cases were divided into two groups according to the presence or absence of recurrent fractures (non-recurrent fractures refer to cases where no fracture occurred within at least 3 months of follow-up after the initial fracture surgery). The gender, age, and imaging indicators of patients in the two groups were collected and measured, including the thoracolumbar (T11-L2) Cobb angle, Cobb angle of the fractured vertebra, height recovery rate of the fractured vertebra, bone cement distribution types, vertebral CT value at three time points: at the time of initial fracture, immediately after surgery, and at the time of recurrent fracture. The influencing factors of recurrent OVCF were explored through inter-group comparative analysis. The measurement methods for imaging data are as follows: (1) Thoracolumbar Cobb angle (from the upper endplate of T11 to the lower endplate of L2), which reflects changes in local force lines; (2) Cobb angle of the fractured vertebra, referring to the angle formed by the fractured vertebra and the intervertebral discs above and below it; (3) Height of the fractured vertebra, referring to the average height of the midpoints of the upper and lower endplates of the adjacent non-compressed vertebrae above and below the fractured one, which is used to calculate the height loss rate and the height recovery rate of the surgically treated vertebra; (4) CT value, referring to the average value calculated after measuring the CT values of the cancellous bone parts of the adjacent upper and lower vertebrae of the fractured vertebra on the median sagittal plane of CT, in the three-dimensional reconstructed CT images, the mid-layer axial view of the vertebral body was selected and a region of interest (ROI) as large as possible was placed in the anterior part of the vertebral trabecular bone on the axial image, avoiding the cortical bone, areas of local hyperostosis, degenerative structures, and the posterior venous plexus. The average Hounsfield Unit (HU) value within the ROI was automatically read using the PACS software routinely employed for image review, representing the CT value of the fractured vertebra; (5) bone cement distribution types,①On anteroposterior view(AP view) X-ray images, the vertebral body was divided into 4 zones by two vertical lines: one along the medial border of each pedicle and one through the vertebral midline. Bone cement distribution patterns were classified as follows: Type I (Diffuse distribution): Cement distributed across zones 1–4, Type II (Central distribution): Cement confined to zones 2 and 3, Type III (Peripheral distribution): Cement located only in zone 1 or zone 4, Type IV (Eccentric distribution): Cement distributed in zones 1–2 or zones 3–4 (Fig. 1);②On lateral view(LAT view) X-ray images, cement distribution was categorized based on its contact with the endplates: Type A: Cement in contact with both the superior and inferior endplates, Type B: Cement in contact with either the superior or the inferior endplate, Type C: Cement not in contact with either endplate.

### Statistical analysis

**Fig. 2:**
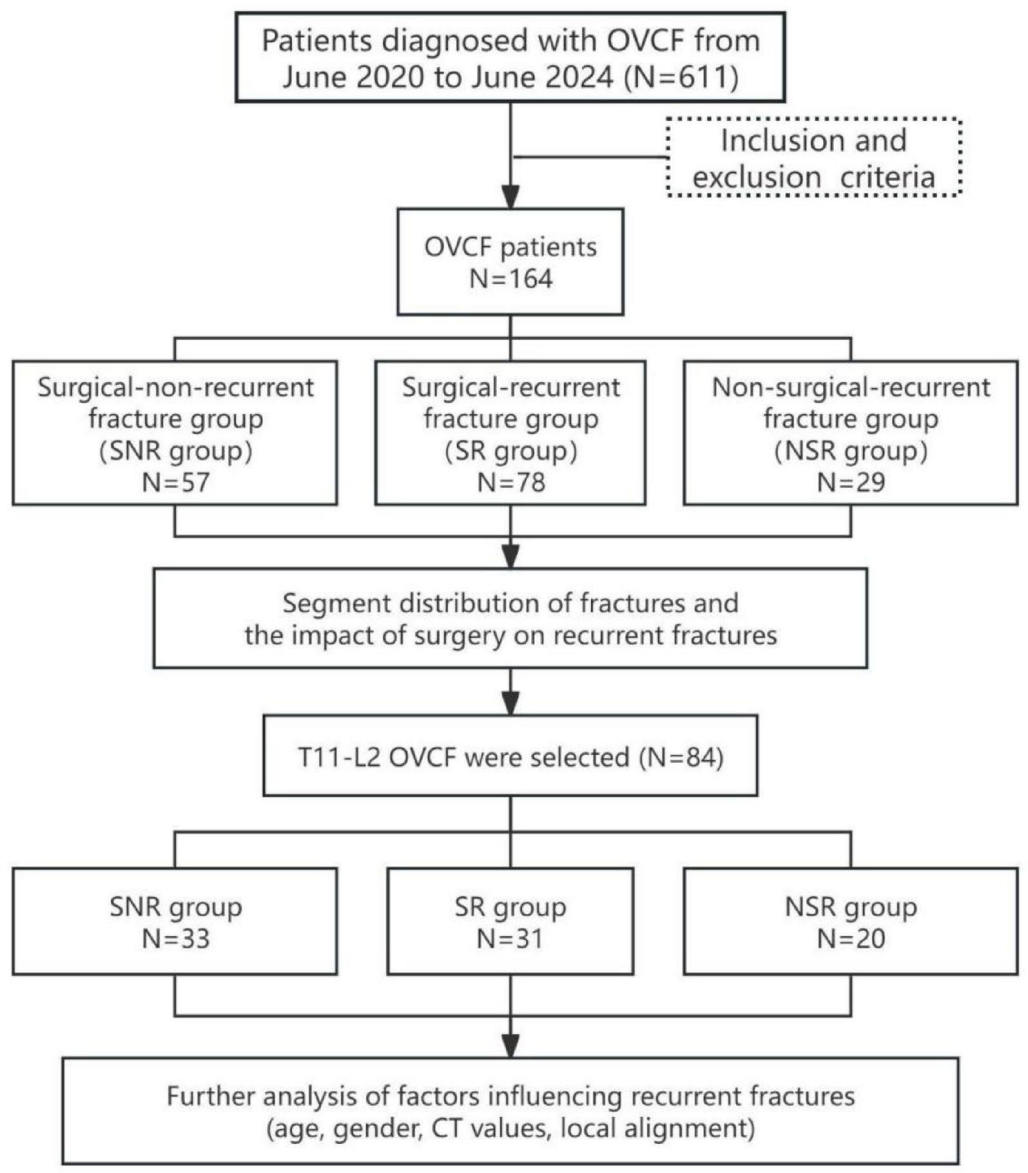
Flow chart of the study

SPSS 27.0 statistical software was used for data analysis, and Graphpad Prism 9.5 was used for graphing. For quantitative data:

Those conforming to a normal distribution were expressed as mean ± standard deviation; Those not conforming to a normal distribution were expressed as median (interquartile range). The chi-square test with the Monte Carlo method was used to compare differences in segmental distribution between groups. For comparisons of quantitative data, either analysis of variance (ANOVA) or nonparametric tests were applied. Logistic regression analysis was used for regression, and receiver operating characteristic (ROC) curves were plotted. The significance level was set at P ≤ 0.05. Flow chart of the study (Fig. 2)

### Results

**Fig. 3:**
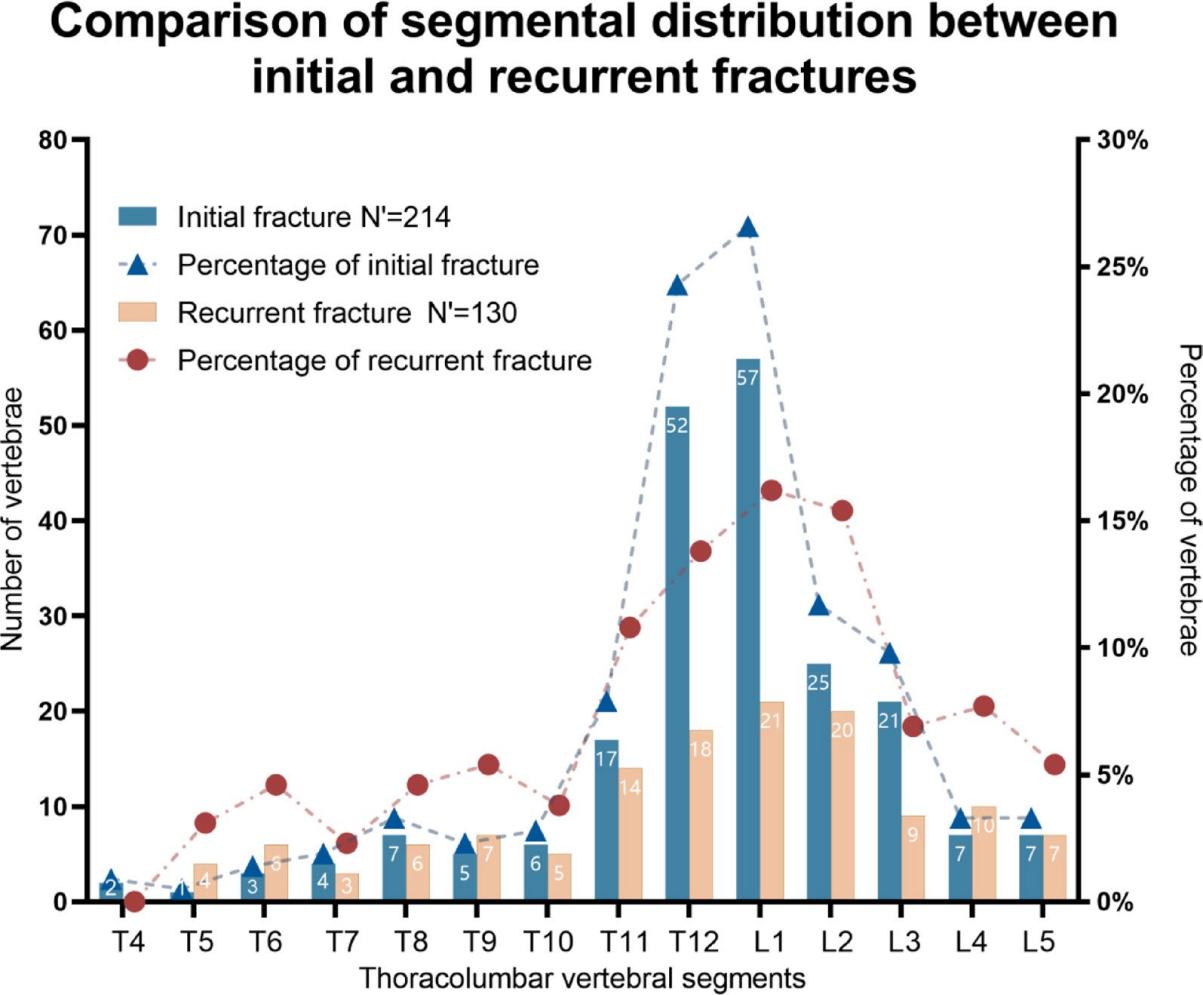
Comparison of segmental distribution between initial fracture and recurrent fracture. *Note*: N': number of vertebrae

2.1 Among the 164 patients in this study, there were 214 vertebrae with initial fractures and 130 vertebrae with recurrent fractures. Both initial and recurrent fractures mainly occurred in the T11-L3 segment. For initial fractures, the highest incidence was at the L1 level, accounting for 26.6% (57 vertebrae), followed by the T12 level, accounting for 24.3% (52 vertebrae); the incidence in the T11-L3 segment reached 80.4%. For recurrent fractures, the highest incidence was also at the L1 level, accounting for 16.2% (21 vertebrae), followed by the L2 level, accounting for 15.4% (20 vertebrae); the incidence in the T11-L3 segment was 63.1% (Fig. 3).

**Fig. 4:**
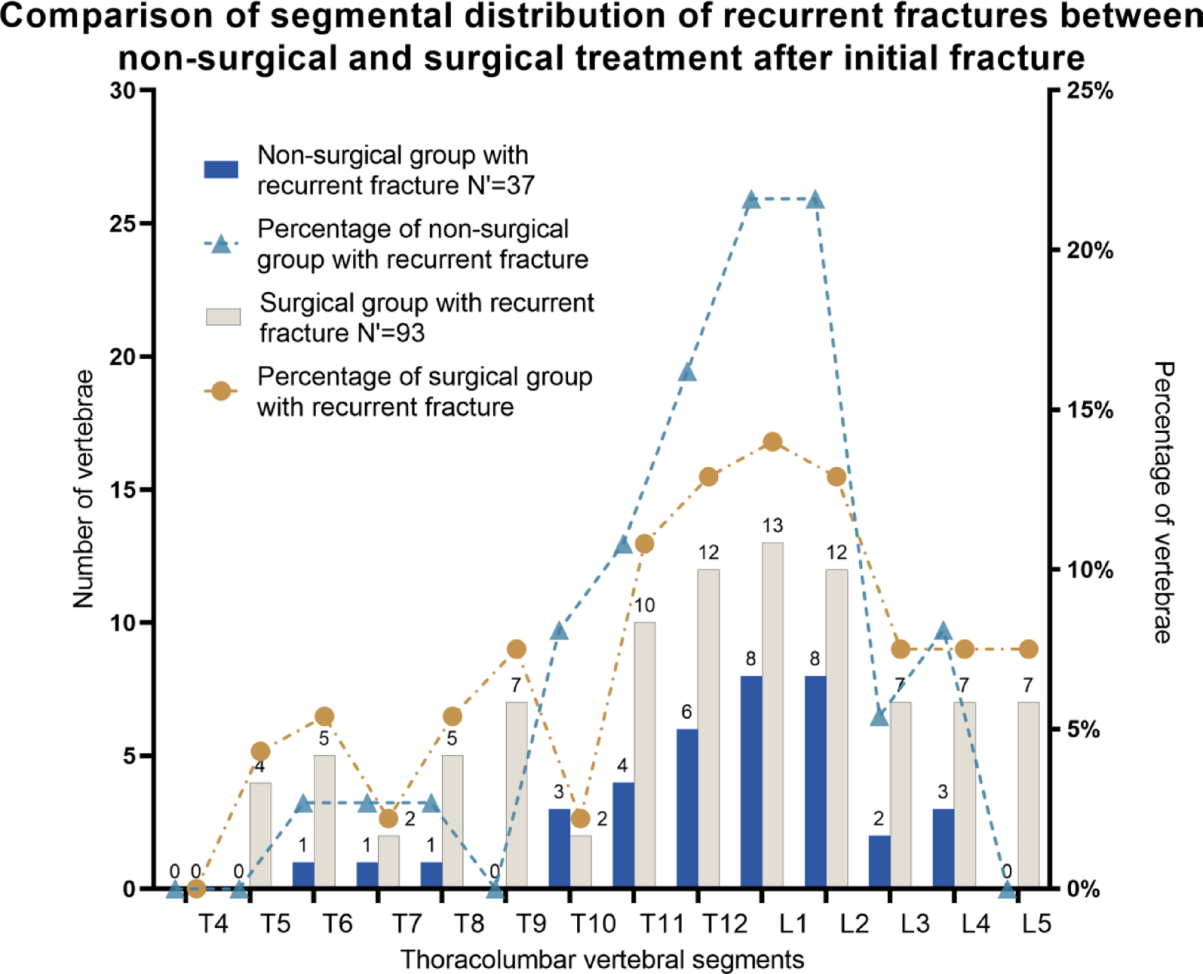
Comparison of segmental distribution of recurrent fractures between non-surgical treatment and surgical treatment after initial fracture. *Note*: N': number of vertebrae

2.2 In the non-surgical treatment group, the highest incidence of recurrent fractures was observed at the L1 and L2 levels, both accounting for 21.6% (8 vertebrae each), followed by the T12 level (16.2%, 6 vertebrae). In the surgical treatment group, the highest incidence of recurrent fractures was at the L1 level, accounting for 14.0% (13 vertebrae), followed by the T12 and L2 level (12.9%, 12 vertebrae each). The chi-square test with the Monte Carlo method was used for analysis, and no significant inter-group differences were found in the incidence and segmental distribution of recurrent fractures between the non-surgical treatment group and the surgical treatment group (χ^2^ = 14.629, *P* = 0.331, Cramér's V = 0.258) (Fig. 4).

2.3 In-depth research was conducted on 84 patients with thoracolumbar OVCF (T11-L2). Two physicians independently conducted the measurements for imaging data. Inter-rater reliability, assessed using the intraclass correlation coefficient (ICC) under a two-way mixed-effects model for consistency, was excellent (single-measure ICC = 0.822, 95%CI: 0.719–0.890; average-measure ICC = 0.902, 95% CI 0.836–0.942; F(59,59) = 10.233, *P* < 0.001). The following results were obtained. Firstly, among the causes of initial and recurrent fractures, falls accounted for the highest proportion (58.3% for initial fractures, 41.2% for recurrent fractures). A history of trauma was reported in 78.6% of patients with initial fractures and 64.7% of patients with recurrent fractures, while all patients without recurrent fractures denied a history of trauma. The chi-square test with Fisher’s exact probability method showed no significant difference in the distribution of fracture causes between initial and recurrent fractures (χ^2^ = 8.77, *P* = 0.06) (Table 1).

**Table 1:** Causes of initial and recurrent fractures in patients with T11-L2 OVCF

Subgroups\Causes of fracture	Trauma				Uncomplained trauma
	Lifting	Bending	Sprain	Falls	
Initial fracture (N = 84)	12 (14.3%)	3 (3.6%)	2 (2.4%)	49 (58.3%)	18 (21.4%)
Recurrent fractures (N = 51)	4 (7.8%)	3 (5.9%)	5 (9.8%)	21 (41.2%)	18 (35.3%)
Non-recurrent fractures (N = 33)	0	0	0	0	33 (100%)

Secondly, multivariate ANOVA and Kruskal–Wallis H test were used to analyze the risk factors for recurrent fractures, and inter-group differences were found in age and CT value (Table 2).

**Table 2:** Demographic and clinical characteristics of T11-L2OVCF patients

Variables	All N = 84	NSR group N = 20	SR group N = 31	SNR group N = 33	t/$\chi$ *2/F*	*P*-value
Sex						
Male	11 (13.1%)	2 (10.0%)	4 (12.9%)	5 (15.2%)	0.292	0.921
Female	73 (86.9%)	18 (90.0%)	24 (87.1%)	28 (84.8%)		
Age (years)	76 ± 9	76 ± 9	80 ± 7	72 ± 9	8.060	< 0.001^***^
Cobb angle of the initial injured vertebrae (°)	3.3(10.5)	4.4 ± 11.5	1.0 ± 8.7	3.4 ± 8.4	0.883	0.417
Cobb angle of thoracolumbar segment (°)	18.0 ± 10.2	19.7 ± 13.2	17.8 ± 7.9	17.3 ± 10.4	0.298	0.862
CT value (HU)	88.6 ± 33.5	74.6 ± 25.7	81.2 ± 26.8	104.1 ± 37.7	6.888	0.002^**^
Loss of height of injured vertebrae (%)	19.5 ± 24.2	35.2 ± 32.1	16 (17)	19.4 ± 10.3	2.777	0.249
Height recovery rate of injured vertebrae (%)	~	~	16.6 (59)	41.8 ± 38.8	444.5	0.368

*HU* Hounsfield units; *P* < 0.05(*), *P* < 0.01(**), *P* < 0.001(***)

The Mann–Whitney U test was used to compare the differences in bone cement distribution patterns between groups on X‑ray AP and LAT views, and the results indicated no significant difference (*P* > 0.05) (Table 3).

**Table 3:** Comparison of bone cement distribution types between SNR and SR groups

Type		All N = 84	SNR group N = 33	SR group N = 31	Z value	*P* value
AP views	Type I	49	24	25	− 0.952	0.341
	Type II	3	0	3		
	Type III	9	7	2		
	Type IV	3	2	1		
LAT views						
	Type A	33	20	13	− 1.461	0.144
	Type B	26	11	15		
	Type C	5	2	3		

*SNR group* surgical-non-recurrent fracture group, *SR group* surgical-recurrent fracture group, *AP view* anteroposterior view, *LAT view* lateral view

Multiple comparison analysis revealed that among patients with thoracolumbar OVCF who underwent surgery, the average age of the recurrent fracture group was 8.4 years higher than that of the non-recurrent fracture group (*P* < 0.001); the average CT value of the surgical-non-recurrent fracture group was 29.5 HU higher than that of the non-surgical-recurrent fracture group (*P* = 0.004) and 22.9 HU higher than that of the surgical-recurrent fracture group (*P* = 0.012) (Table 4).

**Table 4:** Post-hoc multiple comparisons of age and CT values among the three groups

Variable		Mean difference	*P* value
Age (years)	CR group vs SR group	− 4.2	0.195
	CR group vs SNR group	4.2	0.183
	SR group vs SNR group	8.4	** < **0.001***
CT value (HU)	CR group vs SR group	− 6.6	0.744
	CR group vs SNR group	− 29.5	0.004**
	SR group vs SNR group	− 22.9	0.012*

*P* < 0.05(*), *P* < 0.01(**), *P* < 0.001(***)

Finally, a logistic regression model was constructed using logistic regression to study the predictive probability of influencing factors on recurrent fractures. The odds ratio (OR) for age was 1.071, indicating that for each 1-year increase in age, the risk of recurrent fractures increased by 7.1%; the OR for CT value was 0.979, indicating that for each 1 HU decrease in CT value, the risk of recurrent fractures increased by 2.1% (Table 5). For the overall fit: Likelihood ratio test: χ^2^ = 22.187, *P* < 0.001, indicating the model was effective overall. Hosmer–Lemeshow test: χ^2^ = 4.129, *P* = 0.845, indicating the model had a good fit with no significant difference between the predicted probabilities and the actual observed values.

**Table 5:** Logistic regression dependent variable effect values

	B	S.E	Wald$\chi$^2^	*P*-value	*OR*	95%CI
Sex	− 0.562	0.757	0.550	0.458	0.570	0.129–2.516
Age	0.069	0.033	4.422	0.035*	1.071	1.005–1.142
CT value	− 0.022	0.010	4.933	0.026*	0.979	0.960–0.997
Loss of height of injured vertebrae	2.904	1.449	4.016	0.045*	18.254	1.066–312.631
Constant	− 3.365	2.924	1.325	0.250	0.035	

*P* < 0.05(*)

**Fig. 5:**
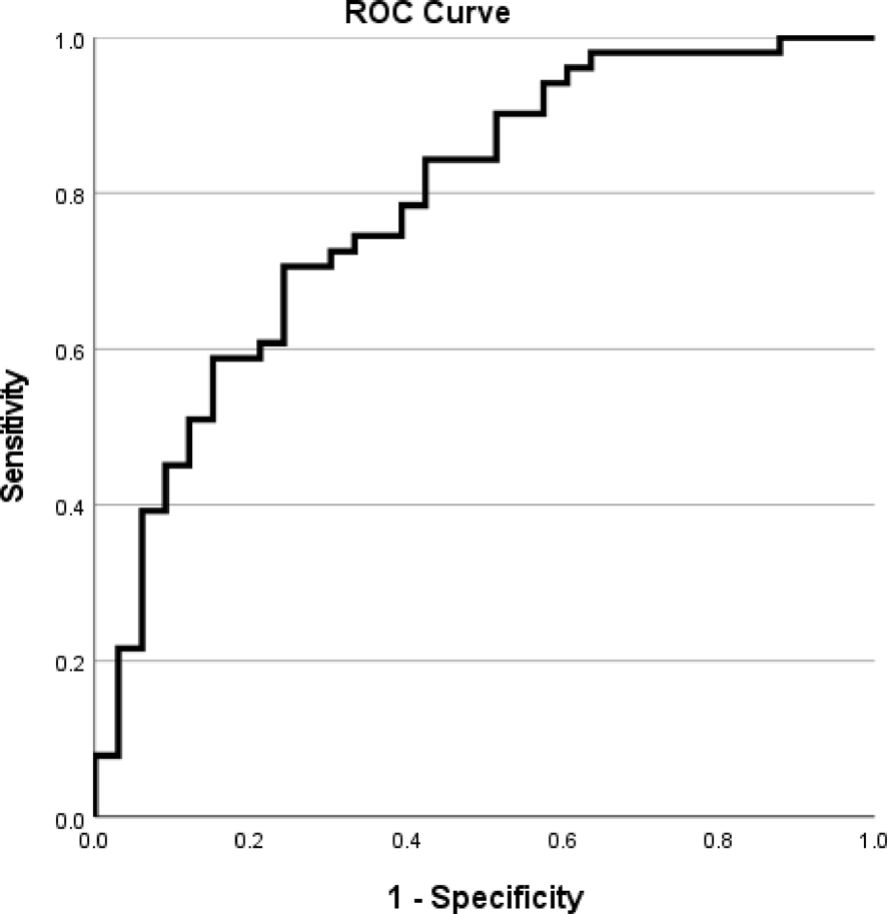
ROC curve. *Note*: The area under the curve (AUC) was 0.786 (95% CI 0.685–0.886, *P* < 0.001), which means the model had moderate predictive ability. For the optimal cut-off value: the maximum Youden index was 0.464, corresponding to a predicted probability cut-off value of 0.65. At this cut-off value, the sensitivity was 70.6% and the specificity was 75.8%

In addition, A receiver operating characteristic (ROC) curve was plotted to evaluate the logistic regression model. The area under the curve (AUC) was 0.786 (95% CI 0.685–0.886, *P* < 0.001), which means the model had moderate predictive ability. For the optimal cut-off value: the maximum Youden index was 0.464, corresponding to a predicted probability cut-off value of 0.65. At this cut-off value, the sensitivity was 70.6% and the specificity was 75.8% (Fig. 5).

## Discussion

Osteoporotic vertebral compression fracture (OVCF) is one of the most common complications of osteoporosis, characterized by severe back pain and limited changes in body position, which leads to a decline in the quality of life. With the increase in the proportion of the elderly population, this clinical issue has attracted growing global attention. Thoracolumbar OVCF is often treated with PVP/PKP surgery, but existing research results on whether surgery affects the occurrence of refracture (recurrent fractures) are inconsistent. A retrospective study by Noguchi T et al. [12] found that approximately 4.9–19% of patients experienced vertebral refractures after surgical treatment, and the probability of postoperative vertebral refractures was the same as that of patients who did not undergo surgery; however, a meta-analysis reported compared with conservative treatment, percutaneous vertebral augmentation increased the risk of postoperative adjacent vertebral fracture [14]. Tang B et al. [15] considered that the closer the vertebral body was to the one treated with PVP surgery, the higher the rate of new fractures. Noguchi T et al. demonstrate that surgery does not increase the overall absolute risk of subsequent fractures at the patient level. The latter discloses that, should a new fracture occur, its distribution is significantly influenced by altered local biomechanics, with adjacent vertebrae becoming relatively more vulnerable due to stress concentration at the vertebral level, while 63.9% of the recurrent fracture still happend at T11-L3 area. We found that whether it was the initial fracture or recurrent fracture, the T11-L3 segment was the most common (75.8% and 63.1%), which was similar to the results of a previous study on 681 cases of OVCF [16]. In that study, the incidence of thoracolumbar (T11-L1) fractures was 56.34%, including T11 (8.49%), T12 (22.61%), and L1 (25.24%). The thoracolumbar transition zone is a high-incidence site for OVCF, which is related to the concentration of biomechanical stress on the spine. As a transition zone between kyphosis and lordosis, it is also a transition zone between mobility and relative stiffness. The vertebral bodies in this zone bear large axial loads and shear forces. Coupled with osteoporosis, even minor external forces can cause fractures in this area [8, 17, 18].

This study analyzed the incidence and segmental distribution of refractures after non-surgical treatment and surgical treatment, and found no significant difference between the two groups, suggesting that whether surgical treatment is performed or not has no clear impact on refractures. The study indicates that both initial and recurrent thoracolumbar OVCF are mostly found in the T11-L3 segment, which is related to the biomechanical characteristics of the thoracolumbar transition zone.

To explore the risk factors for refractures, this study focused on 84 patients with thoracolumbar OVCF for further research. The patients were divided into three groups: surgical-recurrent fracture group, surgical-non-recurrent group, and non-surgical-recurrent fracture group. Gender, age, and imaging data were collected respectively, followed by inter-group comparisons. Multiple comparisons were conducted on factors with significant differences, and it was found that advanced age (> 80 years old) and decreased bone mineral density (CT < 81.2 HU) were independent risk factors for refractures. When constructing the multivariable model, we forced the inclusion of sex and baseline vertebral height loss rate (representing injury severity on top of age and CT value to adequately control for confounding. Logistic regression results showed that for each 1-year increase in age, the risk of refracture increased by 7%, and for each 1 HU decrease in CT value, the risk of refracture increased by 2%. This conclusion is consistent with the findings of some previous studies. For example, Lee et al. [19] found that age > 70 years old and osteoporosis (low lumbar bone mineral density) were significant risk factors for refractures. Multiple studies also pointed out that a bone mineral density (BMD) T-score ≤ -3.5 or a decrease in CT value was significantly associated with the risk of refracture [11, 20]. We noted the *P*-value < 0.05 for the height loss rate, but its 95% confidence interval is extremely wide (1.066–312.631), indicating a highly imprecise effect estimate and unstable result. Therefore, we do not emphasize it as a reliable independent predictor.

In elderly patients, the imbalance of bone metabolism is aggravated, the activity of osteoblasts decreases, and the activity of osteoclasts increases, leading to further loss of bone mass. In addition, elderly patients are often complicated with muscle atrophy and decreased balance function, which increases the risk of falls, thereby accelerating the induction of vertebral refractures [2, 11]. Bone mineral density (BMD) is a key indicator for evaluating the degree of osteoporosis, and dual-energy X-ray absorptiometry (DXA) is the gold standard for diagnosis, but it is greatly affected by bone size, shape, and degeneration [8]. The CT value reflects the degree of X-ray attenuation by bone tissue and is positively correlated with bone mineral density. In this study, the CT value of the recurrent fracture group was significantly lower than that of the non-recurrent group (Δ = 29.5 HU, *P* = 0.004; Δ = 22.9 HU, *P* = 0.012), indicating that patients with refractures had lower bone mineral density and more severe damage to bone microstructure, which was consistent with the pathological mechanism of osteoporosis. In addition, a lot of studies have confirmed that the CT value is significantly associated with the risk of refracture after OVCF surgery [21], [22]. In this study, the average CT values of the recurrent fracture group were 74.6 HU (non-surgical-recurrent) and 81.2 HU (surgical-recurrent), which were lower than the 104.1 HU of the surgical-non-recurrent group, further supporting that a decrease in CT value is a risk factor for refracture. The results of this study suggest that the CT value can be used as a predictive indicator for OVCF refracture and also as one of the simple and effective methods for clinical screening of patients at high risk of refracture.

The mean age of the patients in this study was 76 years, with females accounting for 86.9% of the cohort. All participants were postmenopausal women diagnosed with osteoporosis. Since preoperative bone density examinations were not routinely conducted, CT values were employed in this study to assess the bone density of the patients.Although this study identified vertebral CT value as a significant predictor of recurrent OVCFs, the absence of DXA-measured BMD data represents a notable methodological limitation. DXA remains the gold standard for diagnosing osteoporosis and assessing fracture risk, as recommended by the World Health Organization. CT values are influenced by technical variables such as scanner calibration, tube voltage, and patient body habitus. This may reduce their comparability across different centers and patient populations. Furthermore, DXA provides T-scores that are clinically validated and widely used for therapeutic decision-making, particularly in guiding anti-osteoporosis treatment. Future prospective studies should incorporate both CT attenuation values and DXA measurements to validate their combined or independent predictive value for recurrent fractures.

Surgical treatment can stabilize the fractured vertebral body in the short term, but it cannot reverse the progression of osteoporosis. This suggests that preoperative evaluation is necessary in clinical practice. For elderly patients (especially those over 80 years old) and patients with severe osteoporosis (CT value < 80 HU), it is necessary to fully inform them of the risk of refracture, optimize perioperative management, standardize anti-osteoporosis treatment after surgery, and monitor changes in bone mineral density [23, 24].

Previous studies have shown that early PVP surgery can reduce the risk of long-term deformity, thereby indirectly reducing the probability of refracture [25–27]. However, a meta-analysis suggested factors such as vertebral height recovery rate can increase the risk of adjacent vertebral fractures after surgery [28]. Regarding the question of whether changes in thoracolumbar alignment after OVCF surgery affect refractures, we compared the loss of height of injured vertebrae, height recovery rate of injured vertebrae, thoracolumbar Cobb angle (an imaging parameter) after the initial fracture surgery among the groups. The results showed no significant difference among the three groups. However, due to the small sample size of this study, the association between spinal alignment parameters and refractures may require verification with a larger sample size. Nevertheless, we found that the occurrence of secondary fractures may be related to the following reasons: (1) In patients with severe osteoporosis, osteoporosis plays a dominant role in refractures, and the decrease in overall bone strength may be more critical than local alignment changes; (2) Imaging parameters are affected by body position and projection angle, resulting in measurement errors; (3) Short-term follow-up failed to capture the long-term effects of spinal alignment changes such as progressive kyphotic deformity; (4) The large difference in height loss in the conservative treatment group may mask the effect of the surgical group.

In this study, although the height recovery rate and improvement in local Cobb angle in the surgical group were not statistically significant, their clinical significance may be reflected in pain relief, prevention of aggravated kyphosis, and functional recovery, which help to improve the quality of life of patients. This is consistent with previous studies [10, 13, 29]. This study found that the vertebral height loss rate in the conservative treatment group was higher (35.2%), which may be caused by uncorrected vertebral collapse and large individual differences (with a standard deviation of 32.1%). It may also be related to patient compliance (such as insufficient brace wearing) or differences in the degree of osteoporosis. Patients receiving conservative treatment require long-term immobilization, which may accelerate the progression of osteoporosis [8], and height loss may lead to the progression of kyphotic deformity, eventually forming a "fracture-deformity-refracture" vicious cycle [7, 11]. Therefore, for elderly patients, patients with severe osteoporosis, or patients with poor compliance, early surgical intervention is more advantageous.

The increase in age and the progression of osteoporosis do not mean the absolute occurrence of OVCF. In clinical practice, we followed up some OVCF patients who did not experience secondary fractures after surgery. Combined with previous studies, it is believed that the direct cause of refracture may be closely related to falls or increased load from daily activities [30]. This study reviewed and followed up the cases of thoracolumbar OVCF to determine whether there was a history of trauma before the fracture occurred. Trauma was classified into falls, lifting objects, sprains, and bending over according to different specific mechanisms. The results showed that falls accounted for the highest proportion of causes for both initial and recurrent fractures (58.3% for initial fractures and 41.2% for recurrent fractures). Among patients with initial fractures, 78.6% had a history of trauma, and 64.7% of patients with recurrent fractures had a history of trauma, while all patients without recurrent fractures denied a history of trauma. These results suggest that the occurrence of both initial and recurrent OVCF is directly related to trauma, and the key to preventing OVCF is to prevent trauma such as falls. It is very necessary to evaluate the patient's activity ability and fall risk, and provide balance training and home safety guidance for high-risk patients. We suggest a CT value threshold of < 80 HU as a practical cutoff for identifying patients at high risk of recurrent fracture. Patients identified as high-risk (CT value < 80 HU, age > 80 years) should receive aggressive, combined anti-osteoporosis therapy initiated perioperatively. Regular monitoring of bone turnover markers and DXA BMD every 12–24 months to assess therapeutic response and adherence. Moreover, since 64.7% of recurrent fractures in our cohort were associated with trauma—primarily falls—structured fall prevention programs are essential. These should include: Firstly, home safety assessment with modifications such as removing tripping hazards, installing grab bars, and improving lighting; Secondly, balance and strength training (e.g., Tai Chi, resistance exercises) tailored to elderly capabilities; Thirdly, medication review to minimize psychoactive drugs that increase fall risk; At last, biomechanical education instructing correct posture for lifting heavy objects.

This study has limitations: (1) Retrospective design: There is selection bias. For example, the surgical group may include more patients with acute or severe fractures; (2) Small sample size, especially the conservative group, which may affect statistical power; (3) The refracture rate may be low due to the short follow-up time; (4) Failure to evaluate bone cement parameters such as bone cement distribution type, volume, and leakage, which may affect the results; failure to collect data on bone mineral density measured by DXA; (5) Lack of data on patients who did not experience refractures after conservative treatment; (6) Factors such as female gender and compliance with anti-osteoporosis treatment are also considered independent risk factors for refractures [11, 16, 31, 32]. In this retrospective study, it was also noted that the proportion of females in the refracture group was extremely high (13.1% males vs. 86.9% females). However, due to the limited clinical data records of patients, the status of anti-osteoporosis treatment could not be effectively studied, which can be the focus of follow-up studies.

## Conclusions

Thoracolumbar osteoporotic fractures are mostly found in the T11-L3 segment, and recurrent fractures are also highly prevalent in this area, with no obvious correlation with the location of the initial fracture or whether surgical treatment is performed. Increased age and decreased CT value (low bone mineral density) are risk factors for vertebral refracture. Whether a fracture occurs, whether it is the initial or recurrent one, depends more on the presence of trauma. Therefore, slowing down the decline in bone mineral density and preventing trauma are the keys to preventing thoracolumbar OVCF.

## Data Availability

The datasets used and/or analysed during the current study are available from the corresponding author on reasonable request.

## Abbreviations

OVCF: Osteoporotic vertebral compression fracture
PVP: Percutaneous vertebroplasty
PKP: Percutaneous balloon-expandable kyphoplasty
SNR group: Surgical-non-recurrent fracture group
SR group: Surgical-recurrent fracture group
NSR group: Non-surgical-recurrent fracture group
ROI: Region of interest
AP view: Anteroposterior view
LAT view: Lateral view
ICC: Intraclass correlation coefficient
ROC: Receiver operating characteristic
OR: Odds ratio
AUC: Area under the curve
BMD: Bone mineral density
DXA: Dual-energy X-ray absorptiometry

## References

[1] Koromani F, Li J, Hagino H, et al. The prevention of osteoporotic vertebral fractures in Eastern and in Western countries. Bone Rep. 2025;25:101851. 40495909 10.1016/j.bonr.2025.101851 PMC12148749

[2] Clynes MA, Harvey NC, Curtis EM, et al. The epidemiology of osteoporosis. Br Med Bull. 2020. 10.1093/bmb/ldaa005. 32282039 10.1093/bmb/ldaa005 PMC7115830

[3] Yun X, Zhang L, Fan Z, et al. Global, regional, and national burden of vertebral fractures due to falls from 1990 to 2021 and predictions for the next 15 years: A systematic analysis of the global burden of disease 2021 study. Arch Gerontol Geriatr. 2025;135:105874. 40324317 10.1016/j.archger.2025.105874

[4] Hagino H. Current and future burden of hip and vertebral fractures in Asia. Yonago Acta Med. 2021;64:147–54. 34025188 10.33160/yam.2021.05.001 PMC8128659

[5] Burden AM, Tanaka Y, Xu L, et al. Osteoporosis case ascertainment strategies in european and asian countries: A comparative review. Osteoporos Int. 2021;32:817–29. 33305343 10.1007/s00198-020-05756-8 PMC8043871

[6] Park J-S, Park Y-S. Survival analysis and risk factors of new vertebral fracture after vertebroplasty for osteoporotic vertebral compression fracture. Spine J. 2021;21:1355–61. 33971326 10.1016/j.spinee.2021.04.022

[7] Gutierrez-Gonzalez R, Royuela A, Zamarron A. Vertebral compression fractures: pain relief, progression and new fracture rate comparing vertebral augmentation with brace. BMC Musculoskelet Disord. 2023;24:898. 37980474 10.1186/s12891-023-07041-1 PMC10656983

[8] Yin P, Ma Y, Ma X, et al. The clinical guide line for oste oporotic compre ssion fractures. Chin J Osteoporos. 2015;21(6):643–8 (**In Chinese**).

[9] Chiu P-Y, Kao F-C, Hsieh M-K, et al. A retrospective analysis in 1347 patients undergoing cement augmentation for osteoporotic vertebral compression fracture: Is the sandwich vertebra at a higher risk of further fracture? Neurosurgery. 2021;88:342–8. 33040154 10.1093/neuros/nyaa435

[10] Clark W, Bird P, Gonski P, et al. Safety and efficacy of vertebroplasty for acute painful osteoporotic fractures (VAPOUR): a multicentre, randomised, double-blind, placebo-controlled trial. Lancet. 2016;388:1408–16. 27544377 10.1016/S0140-6736(16)31341-1

[11] Dai C, Liang G, Zhang Y, et al. Risk factors of vertebral re-fracture after PVP or PKP for osteoporotic vertebral compression fractures, especially in Eastern Asia: a systematic review and meta-analysis. J Orthop Surg Res. 2022;17:161. 35279177 10.1186/s13018-022-03038-z PMC8917756

[12] Noguchi T, Yamashita K, Kamei R, et al. Current status and challenges of percutaneous vertebroplasty (PVP). Jpn J Radiol. 2023;41:1–13. 35943687 10.1007/s11604-022-01322-w PMC9813159

[13] Comstock BA, Sitlani CM, Jarvik JG, et al. Investigational Vertebroplasty Safety and Efficacy Trial (INVEST): Patient-reported Outcomes through 1 Year. Radiology. 2013;269:224–31. 23696683 10.1148/radiol.13120821 PMC3781356

[14] Qiu Z, Wang P, Chao Y, et al. The risk of new vertebral fracture after percutaneous vertebral augmentation in patients suffering from single-level osteoporotic vertebral compression fractures: a meta-analysis and systematic review. Medicine (Baltimore). 2023;102:e35749. 37986316 10.1097/MD.0000000000035749 PMC10659685

[15] Tang B, Chen X, Cui L, et al. The closer vicinity to treated vertebrae in percutaneous vertebroplasty, the higher rate of new vertebral compression fractures at follow-up. World Neurosurg. 2024;187:e749–58. 38697261 10.1016/j.wneu.2024.04.162

[16] Chen H, Pan W, Zhang Y, et al. Epidemiological and clinical characteristics analysis of 681 cases of thoracolumbar osteoporotic vertebral compression fractures. Chin J Reparative Reconstruct Surg. 2022;36(7):873–80 (**In Chinese**). 10.7507/1002-1892.202204026 PMC9288900 35848185

[17] Jiang X, Mo L, Liang D, et al. The effect of distribution of bone cement in the fracture lines on the outcome of percutaneous vertebroplasty. Chin J Spine Spinal Cord. 2014;24(2):144–9 (**In Chinese**).

[18] Mao Y, Wu W, Zhang J, et al. Prediction model of adjacent vertebral compression fractures after percutaneous kyphoplasty: a retrospective study. BMJ Open. 2023;13:e064825. 37258076 10.1136/bmjopen-2022-064825 PMC10255151

[19] Lee BG, Choi J-H, Kim D-Y, et al. Risk factors for newly developed osteoporotic vertebral compression fractures following treatment for osteoporotic vertebral compression fractures. Spine J. 2019;19:301–5. 29959099 10.1016/j.spinee.2018.06.347

[20] Li W, Wang H, Dong S, et al. Establishment and validation of a nomogram and web calculator for the risk of new vertebral compression fractures and cement leakage after percutaneous vertebroplasty in patients with osteoporotic vertebral compression fractures. Eur Spine J. 2022;31:1108–21. 34822018 10.1007/s00586-021-07064-z

[21] Wang M, Chen X, Cui W, et al. A computed tomography-based radiomics nomogram for predicting osteoporotic vertebral fractures: a longitudinal study. J Clin Endocrinol Metab. 2023;108:e283–94. 36494103 10.1210/clinem/dgac722

[22] Lee SJ, Graffy PM, Zea RD, et al. Future osteoporotic fracture risk related to lumbar vertebral trabecular attenuation measured at routine body CT. J Bone Miner Res. 2018;33:860–7. 29314261 10.1002/jbmr.3383 PMC5935538

[23] Barnsley J, Buckland G, Chan PE, et al. Pathophysiology and treatment of osteoporosis: challenges for clinical practice in older people. Aging Clin Exp Res. 2021;33:759–73. 33742387 10.1007/s40520-021-01817-y PMC8084810

[24] Jin H, Jin H, Suk K-S, et al. Impact of anti-osteoporosis medication on refracture prevention following osteoporotic vertebral fracture: a systematic review and meta-analysis. Osteoporos Int. 2025;36:2353–79. 40846898 10.1007/s00198-025-07661-4

[25] Takano H, Nojiri H, Shimura A, et al. Early balloon kyphoplasty treatment for osteoporotic vertebral fracture reduces adjacent vertebral fractures. Medicina Kaunas. 2024;60:1097. 39064526 10.3390/medicina60071097 PMC11278625

[26] Liu X, Zhang J, Sun Z, et al. Effects of operation timing of percutaneous vertebral augmentation surgery on treatment of osteoporotic vertebral compression fractures in elderly. J Spinal Surg. 2023;21(1):7–12 (**In Chinese**).

[27] Palmowski Y, Balmer S, Bürger J, et al. Influence of operative timing on the early post-operative radiological and clinical outcome after kyphoplasty. Eur Spine J. 2020;29:2560–7. 32556626 10.1007/s00586-020-06491-8

[28] He L, Li W, Zhai X, Li Z. Risk factors for adjacent vertebral fracture after kyphoplasty or percutaneous vertebroplasty in osteoporotic vertebral systematic review and meta-analysis compression fractures. Eur Spine J. 2025;34:5126–41. 40658233 10.1007/s00586-025-09111-5

[29] Carli D, Venmans A, Lodder P, et al. Vertebroplasty versus active control intervention for chronic osteoporotic vertebral compression fractures: the VERTOS V randomized controlled trial. Radiology. 2023;308:e222535. 37462495 10.1148/radiol.222535

[30] Xia W, Liu Q, Lv J, et al. Prevalent vertebral fractures among urban-dwelling Chinese postmenopausal women: a population-based, randomized-sampling, cross-sectional study. Arch Osteoporos. 2022;17:120. 36070158 10.1007/s11657-022-01158-x PMC9452427

[31] Shi Z, Fan M, Wang Q, et al. Establishing a prediction model for post-operative refracture of osteoporotic vertebral compression fractures. J Pract Orthopaedics. 2024;30(3):205–10 (**In Chinese**).

[32] Yang X, Pi W, Li S. Risk factor analysis for new fractures of other vertebrae after percutaneous vertebroplasty. Chongqing Med J 1–10. (**In Chinese**)

